# Models in the delivery of depression care: A systematic review of randomised and controlled intervention trials

**DOI:** 10.1186/1471-2296-9-25

**Published:** 2008-05-05

**Authors:** Helen Christensen, Kathleen M Griffiths, Amelia Gulliver, Dannielle Clack, Marjan Kljakovic, Leanne Wells

**Affiliations:** 1 Centre for Mental Health Research, The Australian National University, Canberra, Australia; 2 The Australian National University Medical School, The Australian National University, Canberra, Australia; 3 Australian General Practice Network, Minter Ellison Building, Canberra, Australia

## Abstract

**Background:**

There is still debate as to which features, types or components of primary care interventions are associated with improved depression outcomes. Previous reviews have focused on components of collaborative care models in general practice settings. This paper aims to determine the effective components of depression care in primary care through a systematic examination of both general practice and community based intervention trials.

**Methods:**

Fifty five randomised and controlled research trials which focused on adults and contained depression outcome measures were identified through PubMed, PsycInfo and the Cochrane Central Register of Controlled Trials databases. Trials were classified according to the components involved in the delivery of treatment, the type of treatment, the primary focus or setting of the study, detailed features of delivery, and the discipline of the professional providing the treatment. The primary outcome measure was significant improvement on the key depression measure.

**Results:**

Components which were found to significantly predict improvement were the revision of professional roles, the provision of a case manager who provided direct feedback and delivered a psychological therapy, and an intervention that incorporated patient preferences into care. Nurse, psychologist and psychiatrist delivered care were effective, but pharmacist delivery was not. Training directed to general practitioners was significantly less successful than interventions that did not have training as the most important intervention. Community interventions were effective.

**Conclusion:**

Case management is important in the provision of care in general practice. Certain community models of care (education programs) have potential while others are not successful in their current form (pharmacist monitoring).

## Background

Depression is a leading cause of disease burden worldwide [1] and an important risk factor for completed suicide. The management of depression is largely conducted in primary care settings, and, while there is agreement that collaborative care delivers better outcomes than non collaborative care, the components of care that are important in this process are still subject to debate [2-4]. Moreover, treatment is more expensive in collaborative formats than in traditional structures [2]. The treatment of depression in community settings has been advocated as one way to reduce costs [3]. However, interventions in these settings are rare, the components that contribute to their success and their relative effectiveness compared to general practice are not known. If effective components can be identified in either general practice or community settings, more streamlined, cost effective, efficient and reconfigured interventions might be achieved in primary care, broadly defined. For the present review, *primary care *is defined as the first direct form of health care a patient receives that is not delivered in a hospital, a specialist clinic operated by a psychiatrist or conducted in an outpatient hospital setting. Using this definition, primary care is deliverable by a doctor, nurse, paramedic, pharmacist, or a health professional in a community setting.

Findings from recent reviews [4] suggest the importance of case managers with mental health training in the delivery of care, and the importance of specialist supervision. As noted by Bower [4], however, there is no error-free method to determine examine 'active ingredients' in complex care settings, although meta-regression methods, randomised controlled trials manipulating 'active ingredients' and qualitative analysis are all likely to contribute to this understanding. Previous reviews have sought to identify "active ingredients" of care in general practice settings but have not incorporated a broader range of community options [4]. The present review extends previous work through the inclusion of trials based in community based settings.

Components of care (that is the ingredients that make up the package of care provided to patients) have been catalogued in different ways that have not always been carefully defined by researchers, using a variety of terms such as care continuity, provider feedback, and patient education. The imprecision in terms is complicated further by the fact that actions or components of care reflect the broader country-specific health care delivery system. USA-based studies often have an emphasis on managed care, the UK has an emphasis on public community-based general practice, while Australia provides primary care in private medical or psychological practices. Coding and categorising these different components of care is a major challenge for reviews in this area. To overcome some of these problems of definition, we opted for the pragmatic solution of coding interventions using four systems. The first (i) involved coding on the basis of previously used categorisation systems that have described *components of care*. We used terms previously developed by Weingarten [5], Gilbody [3], and Tsai [6]. An example of such a component is provider education, which involves the supply of educational materials or clinical practice guidelines to the treating doctor. The second (ii) involved coding the intervention on the basis of the treatment that was provided. The primary treatments coded were anti-depressant medication, cognitive behaviour therapy (CBT), problem solving, other forms of psychological therapy, including interpersonal psychotherapy (IPT), psychoeducation, combined treatments, including psychological therapy and anti-depressant medication. A third step (iii) was to code on the basis of the primary *intention *or primary aim of the study. For example, if the authors stated that the aim of the paper was to compare the effect of guidelines and education on patient outcomes relative to no guidelines or education, this was coded as a "training and feedback" intervention. The latter coding system allowed us to examine those aspects of care that the authors considered might augment their versions of "standard care". Because the standard care [often treatment as usual (TAU)] against which improvements were compared was not itself standard across studies, we coded TAU using the same coding categories described above for the experimental condition. Finally (iv), we developed a new system of coding which involved a *checklist *of discrete components, with a focus on the provision of care management and on consumer preferences in mental health. These provided a more detailed breakdown than the components described above in (i). However, because these components refer specifically to the detailed procedures described in collaborative care/general practice models, this checklist could only be applied to trials in general practice settings [see Additional file 1]. Due to the volume of potential literature, we included randomised controlled trials or quality controlled trials only.

## Methods

### Study selection

**Figure 1:**
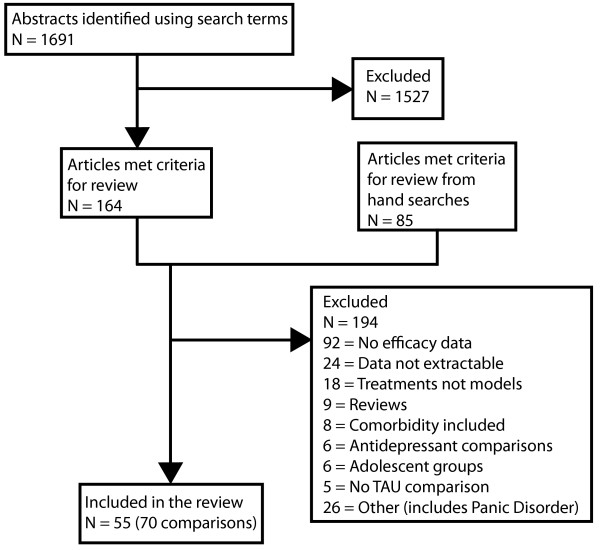
Flow chart of studies for inclusion in the review.

The search which forms the basis of the present review was conducted in October 2005 in PubMed using "Delivery of health care" OR "Patient Health Care" as MeSH terms with "depression" and "trial", and in PsycInfo with "Primary Health Care or Health Care Delivery" and Major Depression as a subject heading. MeSH terms, which are used for indexing PubMed publications, provide a reliable means of capturing papers which use different terms for similar concepts. 1,691 papers were found of which 164 were identified from the abstract description as potential papers for inclusion. An additional 85 papers were located in the reference lists of previous reviews, and by undertaking a search of the Cochrane Library. Search terms "patient care management" OR "delivery of health care" and 'depression" were used to search the Cochrane Central Register of Controlled Trials [See Additional file 2]. The paper with the longest follow-up period was selected for inclusion for studies with more than one published outcome report. The original efficacy or effectiveness trial was sought to replace trials that reported cost effectiveness outcomes, and no publication date restrictions were applied. Figure 1 describes the flow chart for inclusion. 55 studies were identified. Because one study could contain more than one experimental arm, the review undertook an analysis of 70 comparisons. Included in this review were those comparisons where the active enhancement intervention was compared to a control group of either TAU or waitlist control. Studies involving quality and safety issues, side effects, comorbidity or adolescent populations, or published in languages other than English were excluded. To be included, the trial must have contained at least one outcome measure of depression. A summary of the papers included in the review [see Additional file 3] and a quorum statement checklist [see  Additional file 4] are provided.

### Extraction of data

#### Study characteristics

A standard coding sheet was developed, using previous formats with sections based on the Effective Practice and Organisation of Care (EPOC) [7] codes. Three coders (two per paper) coded the following variables: Country, type of control group (TAU, attention placebo, waitlist control, other model of treatment), comorbidity (social anxiety, panic or agoraphobia, generalised anxiety disorder], type of intervention (promotion, prevention, early intervention, treatment, recovery), study design (RCT, CCT), setting (family practice, university affiliated primary care clinic, community service provider, community mental health centre, www, pharmacy, work, other), recruitment procedures (screening in general practice, medical setting, electoral roll, advertisements, other, time period of the intervention (0–6 weeks, 7–11 weeks, 3–4 months, 5–6 months, 7–9 months, 10–12 months, 13–24 months, more than 24 months), treatment content (antidepressants, CBT, etc.), job description of the individual undertaking the intervention [general practitioner (GP), allied health], age of participants, gender, ethnicity, whether consumers were involved in the design, conduct or interpretation of the study. The length of time to the final post intervention follow-up was also recorded. The quality of research papers was rated according to the EPOC Group's criteria [7]. Ratings were made of masking of allocation, blinding, withdrawals, and performance bias. Discrepancies between coders were resolved through discussion, with a third rater acting as an arbiter if required.

### Content of the intervention

#### Coding of components of care

**Figure 2:**
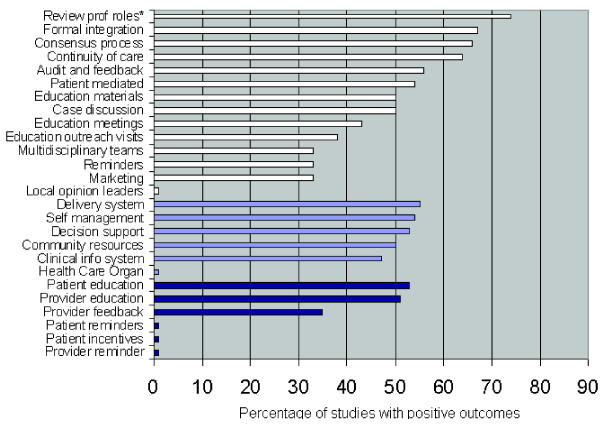
**Percentage of studies reporting positive outcomes as a function of component.** Top white bars refer to components as described by Gilbody (2004), light blue bars to components described by Tsai et al. (2005), and bottom dark blue bars refer to components as described by Weingarten et al. (2002).

We used the classification components outlined in Figure 2 which have previously been used in review of general practice, taking the terms and descriptors from the relevant research articles [3,5,6]. Full descriptions of these categories are available in the original publications.

#### Coding of treatment

Treatment coding was complex because multiple treatments were involved. We sought to determine the most important treatment, or whether combined treatment was used.

#### Coding of intentions

Eight types of intervention were investigated. Most studies focused on only one, but a number of interventions sought to determine the effects of more than one simultaneously. In particular, a number of studies sought to investigate both training and care management, or both enhanced care and care management. For these studies, both relevant categories were scored as positive, and each category of studies was the compared to the rest.

(1) *Training and feedback *directed at general practitioners. These enhancements were provided to general practitioners to improve their skills, and included the components of provider education, the provision of feedback to improve diagnosis, and the provision of clinical practice guidelines.

(2) Providing assistance within the general practice so that patients received '*care management*'. This was achieved variously through the provision of tracking and monitoring by nurses, the provision (and implementation) of procedures designed to encourage adherence to medication or treatment, the use of practice managers to keep patients in care.

(3) *Enhancements or extensions *to general practice care. These involved the use of a specialist involved with the practice, the referral of the patient to health professionals attached to the practice, or the direct provision of enhanced therapy such as problem solving or CBT within the practice.

(4) The provision of *self help *materials or computer guided programs within the practice to improve efficacy.

(5) The provision of assistance from *teams external to the practice *such as mental health teams.

(6) The linking of the patient to *community based health professionals *other than doctors but including pharmacists.

(7) Interventions that occur in *health maintenance *organisations.

(8) Interventions that are initiated in the *broad community *or in smaller facilities within the community, such as educational groups or programs in residential facilities.

#### Detailed component checklist

The checklist included details about the following components: (a) guideline implementation, (b) provider training in depression care, other than by guidelines, (c) patient education including mental health literacy and self help training, (d) the inclusion of patient preferences in the type of care, (e) systematic tracking of patients (other than by the doctor), including details about the nature of the tracking, who does it and whether it is supervised, (f) monitoring of medication adherence, (g) the use of a team based approach, and the nature of the registry or record, (h) additions to usual care provided by the doctor, including enhanced care (CBT, for example), or assistance from another person, including a psychiatrist or social worker, (i) provision of initial patient diagnosis of depression to the GP, and (j) peer support. The full set of questions is provided [see Additional file 1].

### Intervention outcomes

The primary outcome variable was the key depression variable used in the trial. One study which included a quality of life measure was also included [8]. A rating was made of whether there was significant change on that variable relative to the relevant control group (expressed as a dichotomous variable: improved/not improved compared to TAU).

### Analysis

Descriptive data are presented as percentages. Dichotomous outcomes (improved above TAU) were analysed as odds ratios (ORs). Logistic regression was used to predict improvement where multiple components were involved. The Chi Square (*χ*^2^) statistic was used to determine differences in the proportion of positive outcomes as a function of intervention type.

## Results

### Sample characteristics

Papers were published between 1987 and 2005, with 69% from the USA and 21% from the United Kingdom. Almost all studies (91%) compared the intervention to a treatment as usual condition (TAU), while the remaining compared findings to a waitlist control. Ninety three percent of studies were RCTs. Most (61%) were conducted within a general or family practice, with the remainder being conducted in a range of settings, including university primary care clinics (4%), pharmacies (4%) or within the home (14%). The time period over which the intervention took place varied from short term (0–6 weeks, 10%) to more than a year (3%). Most studies provided the intervention over a 3–6 month period (47%). The number of participants ranged from 15 to 2,730 for the active intervention arm. The staff member who provided the intervention consisted of the family doctor (50%), followed by the nurse (26.0%), with psychologist (6%) and pharmacist (6%) being the next largest categories. Most interventions were provided in person (76%), the other major categories being over the telephone (15%), or internet/computer (6%). All employed adult samples, with 21% of studies conducted with older age groups (over 65).

### Quality

In general quality was relatively low for randomised clinical trials. Masking allocation to control for selection bias was clearly stated to have been undertaken by 59% of studies. Blinding of outcome assessments (detection bias) was achieved by 52% of studies, and withdrawal from study (attrition bias) for 64% of studies. Only 5% of studies achieved performance bias standards (providers, recipients, and assessors all blind to assigned intervention).

### Outcome

#### Components of care

Figure 2 describes the percentage of papers reporting positive outcomes as a function of the various components of care using descriptors from previous reviews. Figure 2 ranks the components in order as a function of the percentage of studies with positive outcomes. There was one significant finding. Seventy four percent of studies which included a review of professional roles as an intervention component were associated with improved outcomes over TAU. The odds ratio was 3.8 [CI = 1.08–14.51] indicating a better outcome if professional roles were reviewed.

#### Treatment type

Most interventions used antidepressant medication (AD) (46%), followed by CBT alone (16%), AD combined with CBT (11%) or problem solving/other psychotherapy (10%). Other treatments were psychoeducation (5.7%), problem solving alone (4.3%), 'other psychological therapy' (1.4%) or 'other' (5.7%). We determined whether there was a significant difference in outcomes for the first four categories using the Chi Square statistic. There was no difference [*χ*^2 ^[3, 58] = 3.359, *p *= .340]. The percentages associated with positive outcome were 13/32 (41%) for AD, 6/11 for CBT (55%), 6/8 (77%) for combined treatment, and 4/7 (57%) for AD plus other therapy.

#### Study intentions

Table 1 displays the percentage of studies with a positive outcome relative to TAU as a function of the trial's aim. The significance tests provide an indication of whether interventions coded with the component are more likely to be associated with positive outcomes compared to those without the component. The only feature which was significantly associated with the outcome variable was training and feedback (OR = 8.00), a finding which indicates that this component is significantly *less *likely to result in improved outcomes.

**Table 1:** Number of studies producing an improved outcome as a function of the intervention intention and associated percentage and significance tests.

Intention	Number^a^	Percentage	Exact Sig (2-sided)^b^	Odds Ratio	95% CI Lower	Upper
Training and feedback	2/14	14	.006*	8.00	1.635	39.142
Care management	13/22	59	.31	.538	.193	1.498
Enhancements or extensions	6/15	40	.389	.556	.174	1.775
Self help in general practice	3/6	50	1.00	.939	.176	5.0
Teams external to the practice	0/2	0	-	-	-	-
Community based health professionals	0/3	0	-	-	-	-
Health maintenance	5/8	63	.402	.408	.116	2.401
Broad community.	3/6	50	1.00	.939	.176	5.009

Note: ^a ^Number refers to the number of studies with a positive outcome relative to the number of studies with the intention present. ^b ^Chi-Square. ORs were only undertaken for categories with six or more comparisons.

Table 2 provides findings for the major categories of the detailed component checklist for the 52 comparisons which were examined. Results for subcategories are not displayed unless they are significant. One major category was significant. Trials where patient preferences were taken into account were associated with improved outcomes. Three sub-features of the systematic monitoring category were associated with significant outcomes. When regular scheduled feedback was provided to general practitioners from the person monitoring care, eight of 11 studies were associated with positive outcomes, and these studies were more likely to result in positive outcomes than were studies without this feature [(*χ*^2 ^[1, 52] = 5.289, *p *= .02) (Exact significance 2-sided)]. Similarly, 7 of 7 studies where the 'tracker' provided CBT or problem solving training to the patients were associated with significantly better outcomes [(*χ*^2 ^[1, 52] = 7.544, *p *= .006) (Exact significance 2-sided)]. Provider type also showed a trend to significance for tracking. Of the 22 studies which provided information about the tracker, the following rates of success were attached to different profiles: Nurse 6/11; pharmacist 0/5; psychologist 1/1; psychiatrist 1/1; combination 3/3; other mental health clinician 0/1; or don't know = 1. [(*χ*^2 ^[1, 23] = 12.07, *p *= .06) (Exact significance 2-sided)]. We collapsed the psychologist/psychiatrist, combination and other (mental health clinician) to form a new category 'provider has mental health training' and found a significant difference supporting the superior role of mental health training [(*χ*^2 ^[2, 22] = 7.558, *p *= .021) (Exact significance 2-sided)]. In a series of additional analyses, the nurses were found to be more effective than pharmacists, and mental health professionals more effective than pharmacists. Finally, one sub-feature of the care/prevention plan major category was successful. The care/prevention program was associated with improved outcomes when patient preferences were included (χ^2 ^[1, 52] = 7.54, *p *=.006).

**Table 2:** Number of studies producing an improved outcome as a function of the number of studies in the classification

Component	Number^a^	Percentage	Exact Sig (2-sided)	Odds Ratio	95% CI Lower	Upper
Guideline implementation	9/20	45	.78	.836	.27	2.59
Provider training in depression care, other than guidelines	9/17	53	.37	.53	1.62	1.70
Patient education including mental health literacy and self help training	14/28	50	.11	.50	.16	1.54
Patient preferences incorporated into care	5/5	100	.01*	-	-	-
Systematic monitoring of patients including details about the nature of the tracking, and who does it ^b^	12/22	60	.07	.42	.13	1.29
• Regular scheduled feedback	8/11	72	0.02*			
• Tracker provides CBT	5/5	100	0.01*			
• Provider type	-	-	0.06			
Monitoring of medication adherence	2/3	67	.56	.33	.03	3.87
Team based approach	3/6	50	.30	.70	.13	3.97
Care/prevention plan^b^	10/19	53	.12	.51	.16	1.62
• Includes patient preferences	5/5	100	.01*			
Additions to usual care	3/6	50	1.00	.70	.13	3.87
Provision of initial patient diagnosis of depression	3/11	53	.15	2.30	.53	9.94
Peer support.	0/1	0	1.00	-	-	-

Note: ^a ^Number refers to the number of studies with a positive outcome relative to the number of studies with the intention present. These data based on 52 of the 70 comparisons. Excluded from these analyses were papers that involved community care, psycho-educational interventions in the community, internet interventions, health maintenance organisations, stand alone self help, or one of the trials where more than one contrast or subgroup was included. ^b ^Indicates that a sub category was significant.

To investigate whether the intensity of the intervention influenced outcome, we also created a variable which counted the number of major components from the checklist that were included in the intervention for each study. There was no significant association between a greater number of components and outcome for the trial.

#### The influence of sample size on significance

The sample sizes of the studies ranged from 29 to 4249 (mean 623, SD 839) with approximately 20% of the studies sample sizes over 1000. Where differences exist, studies with larger sample sizes are more likely than smaller studies to find significant differences between intervention and the control conditions. Consequently, it is important to establish that those *Components of Care, Treatments *and *Study Intentions *which were significant were not disproportionately made up of studies with larger sample sizes. Such a result would suggest that significant effects arose because the contributing studies were more sensitive to positive outcomes. We examined this possibility by comparing the sample sizes of studies with the outcomes described above. For *Components of Care *this involved correlating the rank order of the *components *with the average sample size of the contributing studies (both for all studies and then separately for those studies reporting improved outcomes for the intervention over the control condition). We also used scatterplots to determine if systematic associations were present. To illustrate, we ranked the order of the Gilbody components in terms of how well they predicted positive outcomes (highest rank of 1 assigned to 'review of positive roles' and a rank of 14 was assigned to 'local opinion leaders'). We then correlated these ranks with the average size of the samples which found improved outcomes relative to control conditions. For *Treatments *we examined whether a significant difference was present in the size of the samples for the four categories of treatment -AD, CBT, and the two combined therapies. For *Intentions*, we followed the procedure outlined above for *Components*. We also checked whether samples sizes were particularly high for outcomes that had been found to be significant in the above analyses. These analyses yielded essentially negative findings. For the Gilbody Components of Care (see Figure 2) the correlation coefficient of sample size with the rank order of positive outcome was almost zero. Indeed, the full set of analyses failed to find a consistent association between sample size and rank order for *Components of Care, Treatments *and *Study Intentions*. There was no significant difference in sample size between the categories of treatment. In short, we could find no systematic evidence that sample size contributed systematically to significant outcomes.

## Discussion

### Key findings and their relationship with previous literature

Using a variety of approaches to describe the research papers, the current systematic review identified five key outcomes. First, the review found that case management and tracking were associated with improved outcomes for patients with depression. This was demonstrated by the findings that systematic tracking of patients by a provider (other than the doctor) was significantly associated with improved depression outcomes. Where the case manager provided direct feedback to general practitioners and where the case manager provided some form of enhanced care to patients such as the delivery of a psychological therapy (see Table 2) outcomes were significantly better. The revision of professional roles, which typically is brought about by the introduction of the new role of case manager was also found to be associated with improved outcomes (See Figure 2), providing additional support for the importance of systematic tracking. This key finding is supported by previous research [2,5]. Case management may increase adherence and provide the opportunity to deliver enhanced care through the delivery of psychological therapies.

The second key finding was that the monitoring and delivery of treatment was best done by health professionals with a mental health background or by practice nurses rather than by pharmacists. Recent reviews support the importance of a background in mental health [3,4] although other research also supports the contribution of non-mental health trained para-professionals. In these studies, para-professionals delivered chronic disease management as effectively as health professionals, with an overall effect size of .40 being reported [10], see also [11]. One synthesis of these seemingly contradictory findings is that case management improves outcomes above treatment as usual, but that maximum benefit will result if providers are professionally trained in mental health. The reason for this poorer outcome for pharmacists is unclear, but may relate to the general low intensity of the interventions with which they were associated, to non-optimal training of pharmacist for this role or to some other factor.

A third key finding was the significant association between patient preferences and positive depression outcomes. Although the reasons for this association cannot be determined from the present study, previous research in this area has identified that patient preferences improve the likelihood of patients receiving preferred treatments, and of entering treatment [12].

A fourth finding was that the training of general practitioners in depression care and the provision of clinical practice guidelines were not associated significantly with improved outcomes. This concurs with previous research, such as that of Gilbody and colleagues in 2006 [3] which identified that passive provider education or distribution of clinical practice guidelines "have minimal effect on care of depression" (p. 3149). These findings suggest that training and education on their own do not contribute to improved outcomes for patients. This does not imply that interventions would be more effective without these components. Moreover, it is likely that clinician education influences clinical practice, practice organisation, referral mechanisms and team collaboration.

Finally, although few in number, community interventions appeared to offer a level of benefit that appeared to match that of general practice interventions. Examples of community interventions that were associated with positive outcomes in the present review were group psychotherapy programs, recruiting individuals directly from urban and rural communities [13], self referred educational workshops [14], mental health interventions for staff in nursing home environments [15], and interventions conducted in health maintenance organisations [16]. Additional educational programs and internet interventions that were not captured by the search terms used in this review (for example, [17,19]) also provide support for the use of broader community mental health interventions.

### Limitations of the findings

There are a number of limitations to the present study which should be considered when interpreting the findings. First, the search terms used to identify studies "delivering care" may have been unduly restrictive. It has been stated that less than 20% of studies of certain types of primary care medicine are captured in Medline data searches [19]. However, this must be weighed against the potential difficulties that would arise from an over-inclusive search strategy which captured a broad range of outcome research. Our search strategy was a pragmatic decision to review those interventions that were seen by their authors to contribute to the "delivery of mental health care". To complement the current review we also undertook separate reviews of tele-interventions [20], web interventions [21] and school-based prevention programs. Because of this, and the extensive hand searching undertaken we are reasonably confident that we identified the majority of the research literature. Nevertheless, as we only looked at the published literature, publication bias may have influenced the results of the review.

A second criticism is that we provided outcomes in terms of improvement over control rather in the form of an effect size which would indicate the strength of the association, allow us to combine treatment outcomes, and to undertake more sophisticated analyses. Primarily, we chose a categorical outcome measure because we did not want to confer a level of precision that, on the basis of the reviewed literature, was not justified. Moreover, because TAU varied quite markedly across studies, there was no sense of a 'shared' control condition to compare the strength of the association. In this sense the comparisons were relative to a non standard condition. Previous work has reported that a large factor in heterogeneity across studies was the use of different instruments [10].

A major difficulty with any attempts to detect effective components in mental health delivery is that the interventions range from those that were exceptionally complex to those that were relatively unidimensional. As noted by others previously [4], the analysis of complex interventions is difficult, especially when reporting is poor, and when multiple components are included. We found this problem to be almost universal in the present analysis. Even if the intervention arm was described thoroughly, TAU failed to be described in any depth. These difficulties suggest the worthiness of undertaking more focused longitudinal investigations interventions of both general practice and community interventions.

The present review undertook many analyses of multiple comparisons. It is thus possible that our significant findings are due to Type 1 errors. Because study numbers are small the possibility that community and general practice interventions appeared to yield similar levels of outcome may arise simply from low power. Further work is required to compare community and general practice frameworks. Although the checklist for general practice is probably the most comprehensive to date, it is not easily applied to community based trials. Further development of checklists for community based mental health trials are needed.

Another issue concerns the broader interpretation that can be made of the significant findings. Although a number of components were associated with treatment outcomes, these components may be 'proxies' of other unmeasured factors or indeed 'markers' of the study's quality. To illustrate, the inclusion of patient preferences in care may indicate the comprehensiveness of a quality service rather than specifically denoting a causal role for preferences in the production of good depression outcomes.

## Conclusion

The findings from this review point to the importance of case management in general practice environments. They suggest that primary care reform may arise from an emphasis on training in case management skills, the use of structured depression treatment delivered by a case manager in general practice, and through the de-emphasis of general practitioner training.

The other major policy implication from this review is that there is a need to investigate community based mental health care. Evidence is increasing that educational environments – workshops, organisations, and (in the USA) health maintenance organisations are suitable venues to deliver community based care.

## Competing interests

Christensen and Griffiths are the coauthors of MoodGYM, an Internet CBT program and BluePages Depression Information, a psychoeducational website for depression.

## Authors' contributions

HC designed the study, undertook most of the analyses and wrote a draft of the manuscript. KMG designed the study and the searching criteria, developed coding checklists and commented on the analysis and the study. AG and DC undertook the searches, coded the papers, designed the database and commented on the paper. MK and LW assisted in the design of the study and contributed clinical and policy expertise. Both commented on the manuscript. All authors read and approved the final manuscript.

## Pre-publication history

The pre-publication history for this paper can be accessed here:



## Supplementary Material

Additional file 1 Detailed checklist of care provided in general practice and primary care Click here for file

Additional file 2 Search strategy for the identification of studies Click here for file

Additional file 3 Descriptive data for interventions included in the review Click here for file

Additional file 4 Quorum Checklist Click here for file
