# Supplementary material for: Models in the delivery of depression care: A systematic review of randomised and controlled intervention trials
Source: BMC Fam Pract. 2008 May 5;9:25. doi: 10.1186/1471-2296-9-25 (PMC2390560; doi:10.1186/1471-2296-9-25)
Supplement: Additional file 1 — Detailed checklist of care provided in general practice and primary care [file 1471-2296-9-25-S1.doc]

| **#** | **Variable** | **Description** |
| --- | --- | --- |
| 1.1 | Guideline_Implementation | Did the model implement guidelines of any type for patients or providers? |
| 1.2 | ProvWrittenGuide | Were there written guidelines provided to the health provider? |
| 1.3 | ProvFacetoFace | Was the provider provided with face-to-face guideline education? |
| 1.4 | ProvTelephone | Was the provider provided with telephone-based guideline education? |
| 1.5 | PatientWrittenGuide | Did the patient receive written guidelines? |
| 1.6 | PatientFacetoFace | Did the patient receive face-to-face guideline education? |
| 1.7 | PatientTelephone | Did the patients receive telephone guideline education? |
| 1.8 | InvolveCQI_team | Did the model involve the CQI team (organisation-based)? |
| 1.9 | AuditFeedback | Was auditing or feedback provided at the clinican/clinic level? |
| 1.10 | InvolveOpinionLeaders | Were opinion leaders involved in the intervention? |
| 1.11 | MonitorDepStatus | Was depression status measured on more than one occasion? |
| 1.12 | Specific_dep_registry | Was there a specific depression registry/record system? |
| 1.13 | - RecordProviders | Was there a specific depression registry/record system that can be accessed by all providers? |
| 1.14 | - RecordConsumers | Was there a specific depression registry/record system that can be accessed by consumers? |
| 1.15 | - RecordOnline | Was there a specific depression registry/record system online (e-record)? |
| 2.0 | ProviderTrainindepcare | Was there training for the provider in depression care (other than guidelines #1)? |
| 2.1 | GPtraining | Was there GP training? |
| 2.2 | Otherprovider | Was there training provided to other personnel? |
| 3.0 | PatientEducation | Did the intervention include Patient education? |
| 3.1 | EducSymptomsRecognition | Did it include information about symptoms/recognition? |
| 3.2 | EducAetiology_Risk | Did it include information about aetiology/risk factors? |
| 3.3 | EducPrognosis | Did it include information about prognosis? |
| 3.4 | EducBiology | Did it include information about the biology of depression? |
| 3.5 | EducAboutTreat | Did it include information about treatments? |
| 3.6 | - EducAntidep | Did it include information about antidepressant management? |
| 3.7 | - EducTreatmentsOther | Did it include information about treatments other than antidepressants? |
| 3.8 | EducSuicidePrevent | Did it include information about suicide prevention strategies? |
| 3.9 | EducPreventionStrategies | Did it include information about the prevention of recurrence? |
| 3.10 | EducCBT_ProbSolving | Did it include information about CBT/problem solving skills training? |
| 3.11 | EducOtherSelfHelp | Did it include information about other self help training? |
| 3.12 | Other_desc | Did it include any other? |
| 4.0 | PatientPrefsIncorp | Were patient preferences incorporated into care? |
| 5.0 | SystematicMonitor_Patients | Was there systematic monitoring/tracking of patients (by a person other than GP)? |
| 5.1 | Patient_telephone | Was there patient telephone contact? |
| 5.2 | Patient_mail | Was there mail contact? |
| 5.3 | Patient_facetoface | Was there face-to-face contact? |
| 5.4 | TotalContacts | Was the total number of contacts less than 5 or more than 5? |
| 5.5 | RoutineFeedback | Were there routine/regular/scheduled feedback provided to GP on these contacts? |
| 5.6 | SpecificFeedback | Was there specific feedback to flag an issue/problem |
| 5.7 | Spec_dep_registry | Was there a specific depression regisrty/record system |
| 5.8 | - Registry_providers | Was there a specific depression registry/record system that can be accessed by all providers |
| 5.9 | - Registry_consumers | Was there a specific depression registry/record system that can be accessed by consumers |
| 5.10 | - Registry_online | Was there a specific depression registry/record system online (e-record) |
| 5.11 | Provides_pharmacologicalmonitoring | Did the tracking provider provide pharmacological monitoring/advice? |
| 5.12 | Provides_CBT_probsolving | Did the tracking provider provide CBT/problem solving/training? |
| 5.13 | Provides_educational_info | Did the tracking provider provide other educational information? |
| 5.14 | Provides_supportive_counselling | Did the tracking provider provide supportive counseling? |
| 5.15 | Encourages_patientto_fup_withGP | Did the tracking provider encourage patient to f/up with GP? |
| 5.16 | Monitoring_adherenceto_Antidep | Was there monitoring of adherence to antidepressant treatment (e.g. treatment refills)? |
| 5.17 | Monitoring_treat_response | Was there monitoring of treatment response (using formal assessment e.g. questionnaire)? |
| 5.18 | Trackingprovider_referraltoMHcare | Did the tracking provider arrange referral to specialist mental health care? |
| 5.19 | Supervision | Was there supervision provided to tracking provider? |
| 5.20 | Supervisor_type | What was the supervisor type (psychiatrist, psychologist)? |
| 5.21 | Provider_type | What was the provider type? |
| 5.22 | All_Patients | Was tracking provided for all patients? |
| 5.23 | Time_Period | Over what time period? |
| 6.0 | Monitor_meds_adherence | Was their monitoring of meds adherence (eg treatment refills)? |
| 6.1 | TypeofProvider | Who provided the monitoring? |
| 7.0 | Teambased_approach | Was there a team-based approach? |
| 7.1 | FeedbacktoGP | Was there routine/regular/scheduled feedback to GP on contacts? |
| 7.2 | Specific_feedback | Was there specific feedback to flag an issue/problem? |
| 7.3 | Specific_dep_record_system | Was there specific depression registry/record system? |
| 7.4 | All_providers | Was there specific depression registry/record system that can be accessed by all providers? |
| 7.5 | Consumers | Was there specific depression registry/record system that can be accessed by consumers? |
| 7.6 | Online | Was there specific depression registry/record system online (e-record)? |
| 8.0 | care_prevention_plan | Was there a care/prevention plan? |
| 8.1 | Evidence_based | Was it evidence-based? |
| 8.2 | Patient_preference_included | Were patient preferences included? |
| 8.3 | Stepped_care_algorithm | Was there a stepped care algorithm? |
| 9.0 | Additions_to_usualcare_GPplusCBT | Were there additions to the usual care of the GP plus CBT/problem solving? |
| 9.1 | - GP_plus_facetoface | Was the addition face-to-face? |
| 9.2 | - GP_plus_computer | Was the addition computer? |
| 9.3 | - GP_plus_internet | Was the addition internet? |
| 9.4 | - GP_plus_bibliotherapy | Was the addition bibliotherapy? |
| 9.5 | GP_plus_other_psychological_skills_train | GP plus other psychological skills training |
| 9.6 | - face_to_face | face-to-face |
| 9.7 | - computer | computer |
| 9.8 | - internet | internet |
| 9.9 | - bibliotherapy | bibliotherapy |
| 9.10 | GP_plus_socialworker | GP plus social worker? |
| 9.11 | GP_plus_Psychiatrist | GP plus psychiatrist? |
| 9.12 | GP_plus_volunt_comm_orgs | GP plus referral to voluntary community organizations? |
| 9.13 | GP_plus_referral_to_daycare | GP plus referral to day care? |
| 10.0 | Provision_inital_patientdiagnosisofdep_toGP | Was there provision of initial patient diagnosis of depression to GP? |
| 10.1 | GP_provider_consultationadvice | Did the GP provider have access to consultation advice from psychiatrist? |
| 11.1 | Peer_consumer_support | Was there peer (consumer) support/ |
| 11.2 | Peer_support_facetoface | Peer support face-to-face |
| 11.3 | Peer_support_telephone | Peer support Telephone |
| 11.4 | Peer_support_internetbased | Peer support Internet based |
