# Supplementary material for: Models in the delivery of depression care: A systematic review of randomised and controlled intervention trials
Source: BMC Fam Pract. 2008 May 5;9:25. doi: 10.1186/1471-2296-9-25 (PMC2390560; doi:10.1186/1471-2296-9-25)
Supplement: Additional file 2 — Search strategy for the identification of studies [file 1471-2296-9-25-S2.doc]

Trials were identified by electronic searches of PUBMED, PSYCINFO and the Cochrane Central Register of Controlled Trials (Clinical Trials).

The following strategy was used to search PUBMED from 1995-2005 [search conducted December 12, 2005]: Limits: English, Randomized Controlled Trial, Humans, 1995-2005.

#1 Patient Care Management [MeSH] AND “depression” Field: Title/Abstract

#2 Delivery of Health Care [MeSH] AND “depression” Field: Title/Abstract

#3 (#1 OR #2)

The following strategy was used to search PSYCINFO from 1995-2005 [search conducted December 6, 2005]: Limits: 1995-2005.

#1 Primary Health Care [Subject Heading] AND Major Depression [Subject Heading] (search all text)

#2 Health care delivery [Subject Heading] AND Major Depression [Subject Heading] (search all text)

#3 (#1 OR #2)

The following strategy was used to search Cochrane Central Register of Controlled Trials (Clinical Trials) from 1995-2005 [search conducted December 9, 2005].

#1 “patient care management” AND “depression”

#2 “delivery of health care” AND “depression”

#3 #1 OR #2
