# Supplementary material for: Models in the delivery of depression care: A systematic review of randomised and controlled intervention trials
Source: BMC Fam Pract. 2008 May 5;9:25. doi: 10.1186/1471-2296-9-25 (PMC2390560; doi:10.1186/1471-2296-9-25)
Supplement: Additional file 3 — Descriptive data for interventions included in the review [file 1471-2296-9-25-S3.doc]

| **Author** | **Total N *** | **Intervention as defined by authors** | **Outcomes: Intervention improved compared to TAU?** | **Intervention Length** | **Age** | **Final Follow up** |
| --- | --- | --- | --- | --- | --- | --- |
| Arean (2005) | 1801 | Collaborative care treatment model (comparing minority patients) | Yes (all groups) | 10-12 mths | 65+ yrs | 10-12 mths |
| Arthur (2002) | 93 | Follow-up assessment by community mental health team | No | 13-24 mths | 65+ yrs | 13-24 mths |
| Aubert (2003) | 4249 | Telephone counselling and educational materials in a disease management program (505: intervention, 3744: control) | Yes | 7-9mths | adult | 7-9 mths |
| Baker (2001) | 780 | Tailored methods to overcome obstacles to change using psychological theories for primary care providers | Yes | 10-12 mths | adult | 3-4 mths |
| Banerjee (196) | 69 | Intervention by psychogeriatric team in frail elderly people at home | Yes | 5-6 mths | 65+ yrs | 5-6 mths |
| Baumgarten (2002) | 212 | Adult day care for the frail elderly | No | 3-4 mths | 65+ yrs | 3-4 mths |
| Blanchard (1999) | 64 | Brief domiciliary-based research nurse intervention in older people | No | 3-4 mths | 65+ yrs | 13-24 mths |
| Brook (2005) | 147 | Pharmacy-based coaching program for antidepressants in patients | No | 5-6 mths | adult | 5-6 mths |
| Brown (2000) | 928 | Continuous quality improvement | No (all groups) | 0-6 wks | adult | 3-4 mths |
| Brown (2004) | 120 | Psycho-educational self-confidence workshops | Yes | 10-12 mths | adult | 10-12 mths |
| Bruce (2004) | 598 | Primary care intervention on suicidal ideation and depression in older patients (PROSPECT intervention) | Yes | 10-12 mths | 65+ yrs | 10-12 mths |
| Callahan (1994) | 175 | Patient-specific treatment recommendations for primary care providers | No | 3-4 mths | 65+ yrs | 7-9 mths |
| Capoccia (2004) | 74 | Pharmacist enhanced care intervention | No | 10-12 mths | adult | 10-12 mths |
| Clarke (2005) | 299 | Internet depression skills intervention program (ODIN) | No | 7-9mths | adult | 7-9 mths |
| Coleman (1999) | 265 | "Chronic Care Clinics" a new model of primary care for frail older adults | No | 10-12 mths | 65+ yrs | 13-24 mths |
| Corney (1987) | 80 | Social work referral intervention | No | 5-6 mths | adult | 10-12 mths |
| Datto (2003) | 61 | Telephone disease management | Yes | 7-11 wks | adult | 3-4 mths |
| Dietrich (2004) | 405 | Evidence-based care with quality improvement support (telephone support) | Yes | 5-6 mths | adult | 5-6 mths |
| Dowrick (2000) | 452 | Problem-solving treatment and group psychoeducation | Supportive counselling and psychoeducation = No, Problem-solving = Yes | 7-11 wks | adult | 10-12 mths |
| Finley (2003) | 125 | Collaborative care model with clinical pharmacists | No | 5-6 mths | adult | 5-6 mths |
| Fletcher (2005) | 30 | Non-guided self-help manual in primary care | No | 3-4 mths | adult | 3-4 mths |
| Grant (2000) | 161 | Referrals facilitator between primary care and the voluntary sector | No | 3-4 mths | adult | 3-4 mths |
| Hegel (2005) | 1801 | Collaborative care treatment model | Yes (all groups) | 10-12 mths | 65+ yrs | 10-12 mths |
| Holdsworth (1996) | 106 | New self-help manual in primary care | No | 3-4 mths | adult | 3-4 mths |
| Hunkeler (2000) | 302 | Nurse telehealth care and peer support | Nurse telecare plus peer support = No, Nurse telecare = Yes | 5-6 mths | adult | 5-6 mths |
| Jorm (2003) | 1094 | Evidence-based consumer guide on effectiveness of treatment options for depression | No | 5-6 mths | adult | 5-6 mths |
| Katon (2002) | 228 | Collaborative care intervention (Long-term effects) | High depression severity = No, Moderate depression severity = Yes | 3-4 mths | adult | 24 mths + |
| Katon (2001) | 386 | Relapse prevention intervention | Yes | 10-12 mths | adult | 10-12 mths |
| Katon (1995) | 217 | Multifaceted intervention program | Yes (all groups) | 7-9mths | adult | 7-9 mths |
| Lin (1999) | 1999 | Collaborative care (long term follow up) | No | 3-4 mths | adult | 13-24 mths |
| Llewellyn-Jones (1999) | 220 | Multifaceted shared care for late life depression in residential care | Yes | 7-9mths | 65+ yrs | 10-12 mths |
| Lynch (1997) | 29 | Telephone-based problem-solving intervention | No | 0-6 wks | adult | 0-6 wks |
| Lynch (2004) | 54 | Telephone-based treatment problem-solving therapy | Yes | 0-6 wks | adult | 0-6 wks |
| Mann (1998) | 577 | Practice nurse follow-up care | No (all groups) | 3-4 mths | adult | 5-6 mths |
| Miranda (2003) | 267 | Guideline-based care (antidepressants/psychotherapy/community mental health services) for low income minority women | Yes (all groups) | 5-6 mths | adult | 10-12 mths |
| Miranda (2004) | 1356 | Quality improvement interventions aimed at increasing guideline-concordant depression care | Yes | 5-6 mths | adult | 5-6 mths |
| Patten (2003) | 786 | Psychoeducational computer program | No | 0-6 wks | adult | 3-4 mths |
| Proudfoot (2004) | 274 | Computerised cognitive behavioual therapy in primary care | Yes | 7-11 wks | adult | 5-6 mths |
| Richards (2003) | 139 | Supervised self-help cognitive behavioural therapy in primary care (PHASE) | No | 3-4 mths | adult | 3-4 mths |
| Rickles (2005) | 63 | Pharmacist telemonitoring of antidepressant use | No | 3-4 mths | adult | 3-4 mths |
| Rollman (2002) | 200 | Computerised decision support for primary care providers | No (all groups) | 5-6 mths | adult | 5-6 mths |
| Rost (2001) | 479 | Redefinition of primary care roles (QuEST Intervention) | Yes | 5-6 mths | adult | 5-6 mths |
| Sherbourne (2001) | 1299 | Short-term quality improvement intervention | Quality Improvement antidepressants = No, Quality improvement CBT = Yes | 10-12 mths | adult | 13-24 mths |
| Simon | 600 | Telephone care management vs. telephone care management plus telephone psychotherapy | Top of Form  Feedback plus care management = Yes, Feedback only = No | 0-6 wks | adult | 5-6 mths |
| Simon (2000) | 613 | Telephone monitoring, feedback, and management of care | Telephone care management program = No, Telephone psychotherapy program = Yes | 5-6 mths | adult | 5-6 mths |
| Solberg (2001) | 257 | Continuous quality improvement | No | 10-12 mths | adult | 10-12 mths |
| Swindle (2003) | 268 | Integration of specialist (clinical nurse specialists) and generalist care (PRIME-MD) | No | 3-4 mths | adult | 10-12 mths |
| Thompson (2000) | 4192 | Clinical-practice guideline and practice-based education for primary care providers | No | 0-6 wks | adult | 5-6 mths |
| Thulesius (2002) | 460 | Learner-centred education in end-of-life care | Yes | 10-12 mths | adult | 10-12 mths |
| Toseland (2004) | 105 | Structured health education program (HEP) for spouses and frail older adults | Yes | 10-12 mths | 65+ yrs | 10-12 mths |
| Trivedi (2004) | 350 | Algorithm-guided treatment (Texas Medication Algorithm Project) | Yes | 10-12 mths | adult | 10-12 mths |
| Tutty (2000) | 122 | Telephone counselling as an adjunct to antidepressants | Yes | 3-4 mths | adult | 5-6 mths |
| Whooley (2000) | 2346 | Case-finding in elderly patients | No | 24 mths + | 65+ yrs | 24 mths + |
| Worrall (1999) | 42 | Educational strategy on clinical practice guidelines for primary care providers | No | 5-6 mths | adult | 5-6 mths |
| Wright (2005) | 45 | Computer-assisted cognitive therapy | Yes (all groups) | 7-11 wks | adult | 5-6 mths |

Note: * as reported by authors
