# Supplementary material for: Models in the delivery of depression care: A systematic review of randomised and controlled intervention trials
Source: BMC Fam Pract. 2008 May 5;9:25. doi: 10.1186/1471-2296-9-25 (PMC2390560; doi:10.1186/1471-2296-9-25)
Supplement: Additional file 4 — Quorum Checklist [file 1471-2296-9-25-S4.doc]

|  |
| --- |
| | **Heading** | **Subheading** | | **Descriptor** | **Reported? [Y/N]** | **Page Number** | | --- | --- | --- | --- | --- | --- | |  | | | | | | | Title |  | Identify the report as a meta-analysis [or systematic review] of RCTs | | Y | 1 | | Abstract |  | Use a structured format | | Y | 2 | |  |  | **Describe** | | Y | 2 | | Objectives | The clinical question explicitly | | Y | 2 | | Data sources | The databases, i.e. list and other information sources | | Y | 2 | | Review methods | The selection criteria (i.e. population, intervention, outcome, and study design); methods for validity assessment, data abstraction, and study characteristics, and quantitative data synthesis in sufficient detail to permit replication | | Y | 2 | | Results | Characteristics of the RCTs included and excluded; qualitative and quantitative findings (i.e. point estimates and confidence intervals); and subgroup analyses | | Y | 2 | | Conclusion | The main results | | Y | 3 | |  | **Describe** | |  |  | | Introduction |  | The explicit clinical problem, biological rationale for the intervention, and rationale for review | | Y | 4 | | Methods | Searching | The information sources, in detail [e.g. databases, registers, personal files, expert informants, agencies, hand-searching], and any restrictions (years considered, publication status, language of publication) | | Y | 6, 7 | | Selection | The inclusion and exclusion criteria defining population, intervention, principal outcomes, and study design | | Y | 7 | | Validity assessment | The criteria and process used [e.g. masked conditions, quality assessment, and their findings] | | Y | 7, 11 | | Data abstraction | The process or processes used [e.g. completed independently, in duplicate] | | Y | 10 | | Study characteristics | The type of study design, participants' characteristics, details of intervention, outcome definitions, and how clinical heterogeneity was assessed | | Y | 10, 11 | | Quantitative data synthesis | The principal measures of effect [e.g. relative risk], method of combining results (statistical testing and confidence intervals), handling of missing data, how statistical heterogeneity was assessed, a rationale for any a priori sensitivity and subgroup analyses, and any assessment of publication bias | | Y | 12, 13 | | Results | Trial flow | Provide a meta-analysis profile summarising trial flow (see Figure [1](http://mrw.interscience.wiley.com/emrw/9780470011812/eob/article/b2a01049/current/html#b2a01049-fig-0001%23b2a01049-fig-0001)) | | Y | Attached Fig 1 | | Study characteristics | Present descriptive data for each trial [e.g. age, sample size, intervention, dose, duration, follow-up period] | | Y | Attached Table 3 | | Quantitative data synthesis | Report agreement on the selection and validity assessment; present simple summary results (for each treatment group in each trial, for each primary outcome); present data needed to calculate effect sizes and confidence intervals in intention-to-treat analyses (e.g., 2 × 2 tables of counts, means and SDs, proportions) | | Y | 12, 13, 14 | | Discussion |  | Summarise key findings, discuss clinical inferences based on internal and external validity; interpret the results in light of the totality of available evidence; describe potential biases in the review process [e.g. publication bias]: and suggest a future research agenda | | Y | 17, 18, 19 | |
|  |
